# The effects of foliar amino acid and Zn applications on agronomic traits and Zn biofortification in soybean (*Glycine max* L.)

**DOI:** 10.3389/fpls.2024.1382397

**Published:** 2024-04-15

**Authors:** Şule Han, İlker Sönmez, Moin Qureshi, Birgül Güden, Sunil S. Gangurde, Engin Yol

**Affiliations:** ^1^ Department of Soil Science and Plant Nutrition, Faculty of Agriculture, University of Akdeniz, Antalya, Türkiye; ^2^ Department of Field Crops, Faculty of Agriculture, University of Akdeniz, Antalya, Türkiye; ^3^ International Crops Research Institute for the Semi-Arid Tropics, Hyderabad, India

**Keywords:** amino acid, biofortification, mineral nutrition, soybean, zinc

## Abstract

The production and consumption of soybeans are widespread due to their nutritional and industrial value. Nutrient enrichment is vital for improving the nutritional quality of soybeans. This study aimed to evaluate the effect of foliar application of amino acids (AA) and zinc (Zn) on agronomic traits and the accumulation of grain Zn in soybeans. The experimental design comprised 16 treatment combinations involving four levels of amino acid application (0, 50, 100, and 150 ml 100 L^-1^) and Zn (0, 2, 4, and 6 mg L^-1^) following a randomized complete block design with three replications in field conditions. The results demonstrated that the application of foliar Zn and AA did not affect the yield, whereas that of AA_50_*Zn_2_ and AA_150_*Zn_2_ affected the number of pods and branches. The effects of AA application on N and the protein content in grains were determined to be significant. The application of AA_100_*Zn_6_ emerged as the most effective treatment for the enhancement of Zn biofortification in soybean grains. The combined foliar application of AA and Zn contributed to enhanced Zn accumulation in the grains.

## Introduction

Increasing the nutrient density and bioavailability in crops is important for combating hidden hunger and ensuring food security in growing populations. There has been an increase in the number of applications aiming to optimize the utilization of the consumption of goods by living organisms and address shortages of essential nutrients. Organizations such as the World Health Organization (WHO) and the Consultative Group on International Agricultural Research (CGIAR) emphasize the importance of biofortification and prioritize the enrichment of the consumed parts of plant products with amino acids, proteins, vitamin A, and elements such as calcium, magnesium, iron, zinc, copper, selenium, and iodine (Orman and Ok, 2016). Nutrient enrichment studies in plants using agronomic biofortification have gained significant importance in recent years, with the demand for items with elevated nutritional value in the food industry notably increasing. Fundamental nutrition philosophy emphasizes the use of high-quality, nutrient-dense items over quantity in terms of the preservation of living health. Researchers have attempted to address micronutrient deficiencies with different interventions, which may be categorized into four main groups—pharmaceutical supplementation, industrial fortification, dietary diversification, and biofortification (Meenakshi et al., 2010).

The most appropriate method to reduce microelement deficiency is biofortification, which involves the biological enrichment of staple food crops with essential micronutrients. The biofortification process prioritizes the strategies of breeding new cereal genotypes rich in microelements or expanding the use of fertilizers containing microelements (Cakmak, 2008). Agronomic biofortification through fertilization (soil, foliar fertilization, and grain coating) aims to increase the nutrient content of plants without changing their genetic structure (Storksdieck and Hurrell, 2009). The foliar application of mineral fertilizers to plants is an environmentally friendly and cost-effective agronomic strategy for biofortification in an easily phyto-available form (White and Broadley, 2011; Rawat et al., 2013). The efficacy of agronomic biofortification in enhancing zinc (Zn) levels however, a combination of soil and foliar treatment for Zn yields the most favorable results. Zinc plays an important role in the synthesis of tryptophan, the basic component of some important proteins, and with its deficit, plants experience a drop in tryptophan concentration, cessation of protein synthesis, and accumulation of free amino acids (Turan and Horuz, 2012; Acık and Oncan Sumer, 2023). This condition inherently results in reduced yield up to 40% and quality (Noulas et al., 2018). Zn deficiency also causes physiological stress, which results in the development of abnormalities such as stunted growth, chlorosis in leaves, small leaves, and spikelet sterility (Alloway, 2009). Plants become more susceptible to damage from high light intensity and temperature, as well as to infection by certain fungal diseases (Marschner, 1995; Cakmak, 2000).

The use of Zn sulfate is the optimum form for meeting zinc requirements. The timing of foliar Zn application is important; it is generally known to be more effective during the middle phase of root development or in the early milk stage (Lyons and Cakmak, 2012). It is an effective way to improve the concentration of Zn in cereals. The application of 0.5% (w/v) Zn foliar fertilizer at later growth stages of the crop resulted in a greater Zn increase in edible parts, such as grains, indicating that this technique can maximize Zn accumulation (Cakmak et al., 2010). Zinc, a cofactor with structural and catalytic activities in 10% of human proteins (Acık and Oncan Sumer, 2023), plays a crucial role in human health and immune systems (Brown et al., 2004; Sánchez-Palacios et al., 2023). 17% of the global population has insufficient zinc intake (Kumssa et al., 2015), and many individuals do not get enough zinc in their diets (Bouis and Welch, 2010). Prior studies have established that the zinc needed for nutrition can be supplied by using zinc fertilizer in grains (Joy et al., 2015; Wang et al., 2016). Agronomic biofortification through the application of fertilizers that promote the fortification of food crops (especially Zn-containing crops) may be an important strategy in countries with high nutrient deficiency (Joy et al., 2015).

Amino acids (AA) are crucial due to their extensive use in the production of a broad range of chemical molecules, increasing yields and quality, and reducing the productive cycle while improving dry material content (Wahba et al., 2015). They may be used as adjuvants to improve the efficiency of foliar fertilization by increasing the permeability of the leaf cuticle and enhancing nutrient uptake efficiency (Moreira et al., 2015; Moreira and Moraes, 2017).

Soybeans (*Glycine max* L.) are a valuable high-protein source that can help meet human nutrient requirements (Zhan et al., 2019). It has a significant position as a crucial seed legume, accounting for 25% of world vegetable oil output and two-thirds of its protein concentrate used for animal feed (Agarwal et al., 2013). Soybean is well-suited for biofortification because of its elevated protein and oil levels, flexibility as a food and feed component, and adaptability in various conditions. Fertilizer containing zinc (Zn) have been widely accepted as prompt and convenient treatments to address Zn deficiency issues in cereal crops. Although several studies have been conducted on the addition of Zn to different crops focusing on correcting Zn deficiency and increasing grain yield, the majority of the research has focused on yield parameters in cereals. With the HarvestPlus Biofortification Challenge Programme, there is an increasing focus on the biofortification of food crops with Zn using plant breeding (genetic) and agronomic approaches (Lyons and Cakmak, 2012). This study, therefore, aimed to improve the effectiveness of Zn biofortification in soybeans by the combined application of Zn and AA via foliar spraying.

## Materials and methods

### Plant material

The Victoria soybean cultivar was used as the genetic material in this study. This variety has a protein content ranging from 39% to 41%, and an oil content ranging from 18% to 20%. It is additionally characterized by a brown pod color, high potential for yield in secondary crop production, and notable adaptation and tolerance to various diseases, lodging, whitefly infestations, and shedding.

### Site description

The experiment was carried out in a field condition (36°53’54.4”N 30°38’28.9” E) at Akdeniz University, Turkey between June and September 2022. The physical and chemical parameters of the soil samples were analyzed in detail and were obtained at a depth of 0–20 cm in each plot within the experimental area. The total CaCO_3_ (Evliya, 1964), organic matter (Black, 1965), pH (Jackson, 1967), EC (Thomas, 1982), texture (Bouyoucos, 1955), total N (Black, 1957), available P (Olsen and E.L., 1982), extractable K, Ca, and Mg (Kacar, 1972), and available Zn, Mn, and Cu (Lindsay and Norvell, 1978) were determined. Some soil properties (0–20 cm) assessed as follows: loamy in texture, organic matter (1.23%), CaCO_3_ (20.9%), pH = 7.7, EC (0.045%), 0.089% total (N), 4.03 mg kg^-1^ available (P), 96.8 g kg^-1^ extractable potassium (K), 2425.5 g kg^-1^ extractable calcium (Ca) and 146 g kg^-1^ extractable magnesium (Mg), 0.82 mg kg^-1^ available zinc (Zn), 5.51 mg kg^-1^ available manganese (Mn) and 0.88 mg kg^-1^ available copper (Cu) (**Supplementary Table S1**). The mean monthly rainfall, air temperature, and humidity are presented in **Supplementary Figure S1**.

### Experimental parameters: dosages and chemicals

Zinc treatments were applied to the plants as zinc sulfate (ZnSO_4._7H_2_O). The amino acid liquid products comprised total amino acids of 46.70%, total organic matter, organic N, and organic carbon concentrations of 51.35%, 5.42%, and 24.38%, respectively. Amino acids and ZnSO_4_ were applied twice during the BBCH (Biologische Bundesanstalt, Bundessortenamt and Chemical industry) 13 (3 leaf) and BBCH 60-62 stages (beginning of flowering) of soybean plant growth. All amino acids were diluted 100 times with water and sprayed onto the plant leaves each time. The control plants (AA_1_ and Zn_1_) were sprayed with tap water. The chemical composition of the commercial amino acid preparation included glycine (1.45 g 100 g^-1^), alanine (0.25 g 100 g^-1^), valine (0.56 g 100 g^-1^), isoleucine (0.34 g 100 g^-1^), threonine (0.06 g 100 g^-1^), serine (0.14 g 100 g^-1^), lysine (0.28 g 100 g^-1^), phenylalanine (0.23 g 100 g^-1^), glutamate (0.42 g 100 g^-1^), aspartate (0.12 g 100 g^-1^), arginine (0.72 g 100 g^-1^), proline (1.65 g 100 g^-1^), leucine (0.37 g 100 g^-1^), histidine (0.11 g 100 g^-1^), asparagine (0.09 g 100 g^-1^), cystine (0.04 g 100 g^-1^), hydroxyproline (0.78 g 100 g^-1^), methionine (0.45 g 100 g^-1^), tryptophan (0.04 g 100 g^-1^) and tyrosine (0.13 g 100 g^-1^).

### Experimental design

The experiment was performed with 16 treatment combinations involving four levels of amino acids (0, 50, 100, and 150 ml 100 L^-1^ as AA_1_, AA_2_, AA_3,_ and AA_4_, respectively) and Zn (0, 2, 4, and 6 mg L^-1^ as Zn_1_, Zn_2_, Zn_3_ and Zn_4_, respectively), followed by a randomized complete block design with three replicates. The field was plowed twice followed by planking. In terms of basic fertilization, 12 kg da^-1^ NPK (15:15:15) fertilizer was applied to the soil, but the use of zinc was limited to foliar application. Sowing was performed in the second week of June using the Viapora method with a 4–5 cm plant spacing, and row-to-row spacing was maintained at 70 cm. A total of ten soybean grains were planted on each parcel.

### Sample collection

The plant height (cm), number of branches, number of pods, first pod length (cm), and grain yield (g per plant) were also measured in selected plants. The concentrations of nitrogen and zinc were also analyzed in soybean leaves and grains. Leaf samples were taken together with the petiole at the R2 growth stage (Fehr et al., 1971) after 2-week foliar treatments.

Recommended agronomic practices were applied as per crop and climatic parameters. Harvesting was performed manually from the experimental plots after grain hardening (leaf yellowing). In terms of agronomic measurements, five healthy plants were randomly selected from each plot and labeled to determine their developmental characteristics.

For the mineral analysis of the leaf and grain samples, each dried plant and grain sample (0.5 g) was digested with an acid mixture of 10 mL HNO_3_/HClO_4_ (4:1) on a hot plate. The samples were then heated until a clear solution was obtained. This procedure was repeated several times. The concentrations of zinc (Zn) in the digests were determined using inductively coupled plasma (Perkin Elmer Optima DV7000-ICP OES) (Kacar and Inal, 2008). The total nitrogen (N) was determined using the modified Kjeldahl method (Bremmer, 1965).

### Statistical analysis

The data were analyzed using analysis of variance (ANOVA) using SAS statistical software (SAS Institute, 2011). The least significant difference (LSD) test was used at a significance level of p<0.05.

## Results

**Figure 1:**
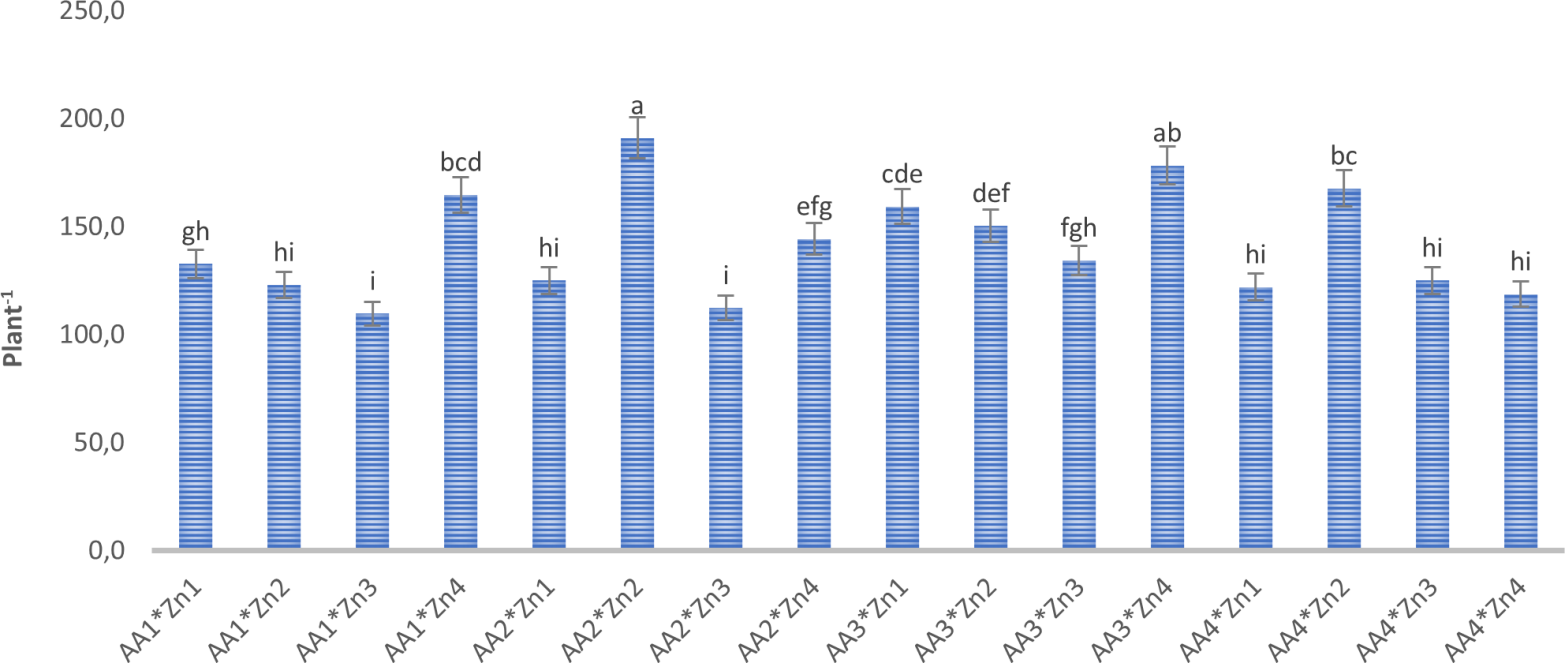
Effect of foliar AA and Zn treatments on number of pods (plant^-1^) of soybeans. The letters on the bars indicate the difference between the mean values.

**Figure 2:**
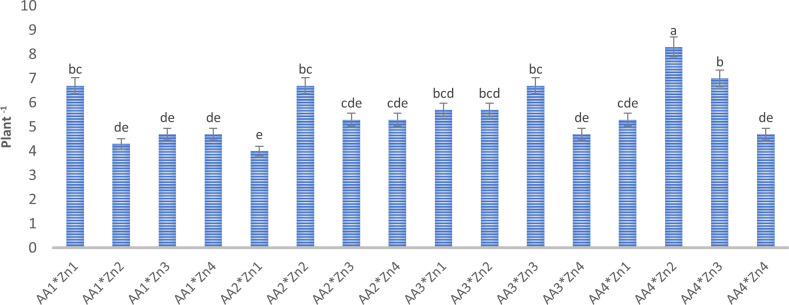
Effect of foliar AA and Zn treatments on number of branch (plant^-1^) of pods of soybeans. The letters on the bars indicate the difference between the mean values.

**Figure 3:**
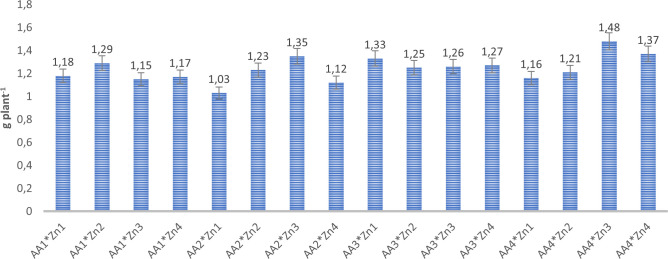
Effect of foliar AA and Zn treatments on grain yield of soybeans.

Foliar doses of AA and Zn significantly increased the average number of pods in soybeans (p<0.001). As shown in **Table 1**, While the highest average number of pods of soybean was obtained from treatment A_3_ (155.6 plant^-1^) in AA treatment dose, the same was observed Zn_2_ (158 plant^-1^) in the Zn treatment dose. The results demonstrated that foliar doses of AA and Zn increased the number of pods in the plants, with the AA × Zn interaction being statistically significant (p<0.001). The highest number of pods was recorded in the AA_2_*Zn_2_ treatment (191 plant^-1^), with no linear relationship between the number of pods and dose increase (**Figure 1**). The effects of foliar AA and Zn treatments on the number of soybean branches are presented in **Table 1** and **Figure 2**. The results indicated that foliar doses of AA and Zn increased the number of branches and that the AA × Zn interaction was statistically significant (p<0.001). The highest number of branches was recorded in the AA_4_*Zn_2_ treatment (8.3 plant^-1^), and foliar treatment doses of AA and Zn significantly increased the average number of branches in soybeans (p<0.001). The highest average value was observed in A_4_ (6.3 plant^-1^) under the AA treatment, and in Zn_2_ (6.3 plant^-1^) under the Zn treatment. Statistical analysis revealed that there was no significant effect on the plant height or first pod length of soybean when treated with AA and Zn. The recorded values for soybean plant height ranged from 110.9 cm to 117.2 cm, whereas the first pod length varied between 15.4 cm and 18.8 cm. The effects of foliar AA and Zn treatments on the grain yield of soybean plants are presented in **Figure 3**. The effects of AA and Zn treatments on grain yield were not found to be statistically significant. The soybean seed yield varied between 1.03 and 1.48 g plant^-1^. There was no significant correlation observed between foliar AA and Zn treatments or grain yields.

**Table 1:** Effect of foliar AA and Zn treatments on average number of pods (NOP), number of branch (NOB), grain yield (GY), plant height (PH) and first capsule lenght (FCL) of soybeans.

Treatments	NOP	NOB	GY	PH	FCL
	(plant^-1^)	(plant^-1^)	(g plant^-1^)	(cm)	(cm)
**AA_1_**	132.5 C	5.1 B	1.19	117.2	16.0
**AA_2_**	143.2 B	5.3 B	1.18	115.5	18.8
**AA_3_**	155.6 A	5.7 B	1.27	110.9	15.9
**AA_4_**	133.3 C	6.3 A	1.30	114.3	17.8
**Zn_1_**	134.8 B	5.4 BC	1.17	113.8	18.4
**Zn_2_**	158.0 A	6.3 A	1.25	114.5	15.4
**Zn_3_**	120.3 C	5.9 AB	1.31	116.3	18.6
**Zn_4_**	151.5 A	4.8 C	1.23	113.2	16.1
**AA**	16.517***	5.828**	1.230ns	1.619ns	0.761ns
**Zn**	41.034***	7.575***	1.055ns	0.430ns	0.984ns
AA*Zn	17.22***	6.962***	0.992ns	1.223ns	0.402ns

The values followed by uppercase letters indicate the difference between the mean values of amino acid treatments. Values followed by uppercase letters in brackets indicate the difference between Zn treatments. ** p<0.01 *** p<0.001 ns, non-significant.

**Figure 4:**
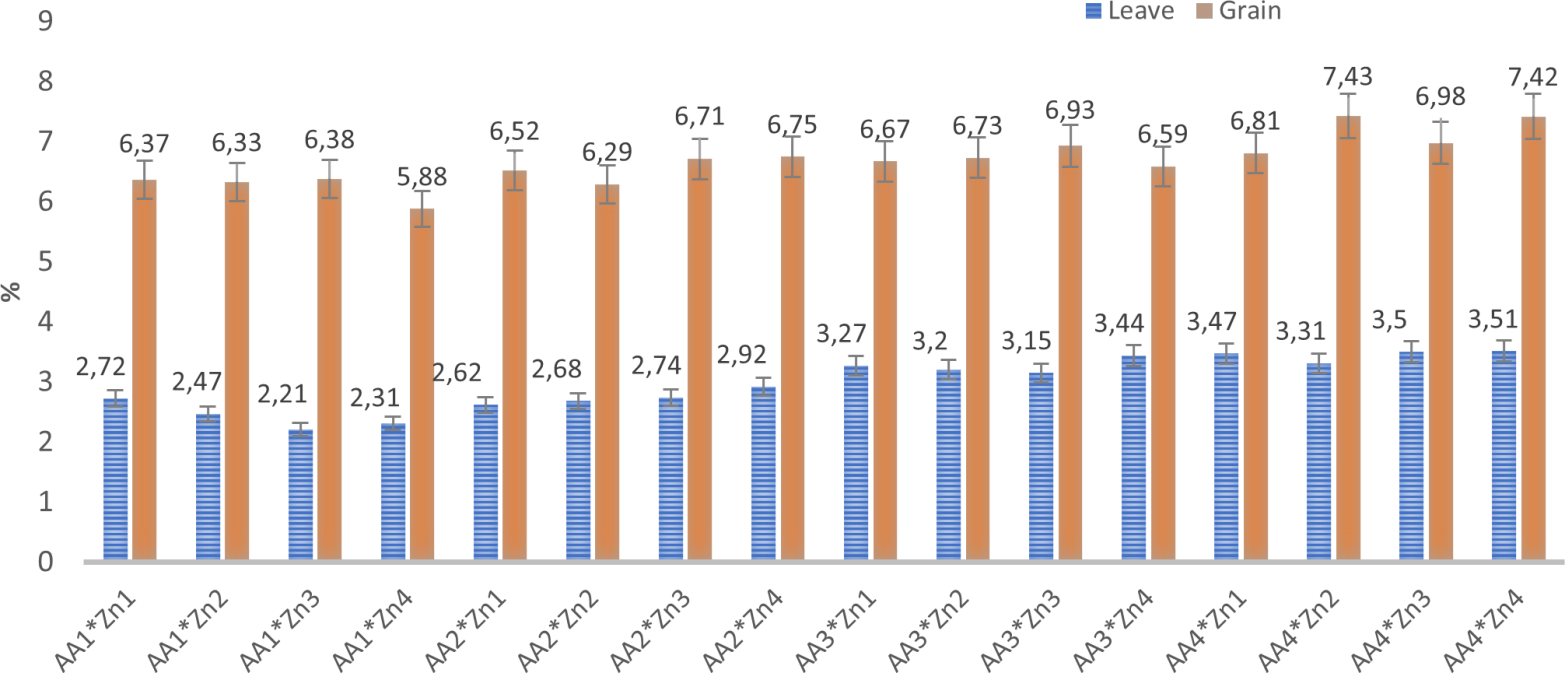
Effect of foliar AA and Zn treatments on N contents (%) of soybeans leaves and grains.

The effects of foliar AA and Zn treatments on the nitrogen content of soybean plants and grains are presented in **Table 2** and **Figure 4**. The findings of this study indicate that foliar spraying with AA and Zn resulted in increased nitrogen levels in soybean plants and their corresponding grains. Foliar treatment with AA and Zn significantly increased the average nitrogen content of soybeans (p<0.001). The highest average nitrogen content was obtained in the A_4_ treatment in soybean leaves (3.45%) and grains (7.16%) with AA treatment doses. Statistical analysis did not reveal any significant interactions between AA and Zn treatments in either the leaves or grains. Furthermore, the application of different Zn dosages did not significantly affect the nitrogen content in the leaves and grains.

**Table 2:** Effect of foliar AA and Zn treatments on average N concentration, Zn concentration and proetin content of soybeans.

Treatments	N concentration		Protein	Zn concentration	
	%		%	mg kg^-1^	
	Leave	Grain	Grain	Leave	Grain
**AA_1_**	2.43 D	6.24 C	39.0 C	26.3 D	48.3 B
**AA_2_**	2.74 C	6.57 B	41.0 B	29.3 C	48.6 B
**AA_3_**	3.27 B	6.73 B	42.1 B	32.2 B	50.5 A
**AA_4_**	3.45 A	7.16 A	44.8 A	35.2 A	51.3 A
**Zn_1_**	3.02	6.60	41.2	27.7 D	40.4 D
**Zn_2_**	2.92	6.69	41.8	30.1 C	47.8 C
**Zn_3_**	2.90	6.75	42.2	31.7 B	51.0 B
**Zn_4_**	3.04	6,66	41.6	33.6 A	59.5 A
**AA**	59.487***	15.886***	15.850***	41.363***	13.667***
**Zn**	1.405ns	0.462ns	0.448ns	98.138***	415.813***
AA*Zn	1.592ns	1.845ns	0.840ns	1.367ns	3.354*

The values followed by uppercase letters indicate the difference between the mean values of amino acid treatments. Values followed by uppercase letters in brackets indicate the difference between Zn treatments. * p<0.05 *** p<0.001 ns, non-significant.

**Figure 5:**
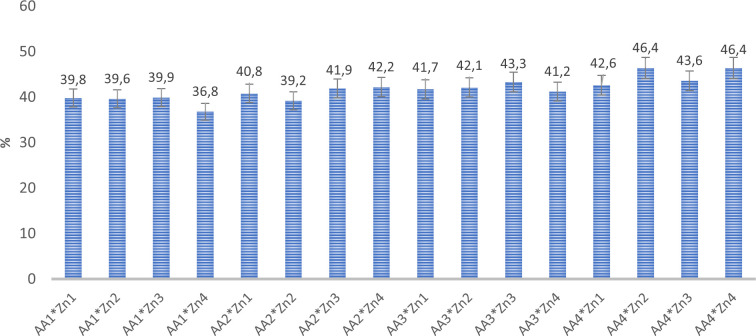
Effect of foliar AA and Zn treatments on protein contents (%) of soybeans grains.

**Figure 6:**
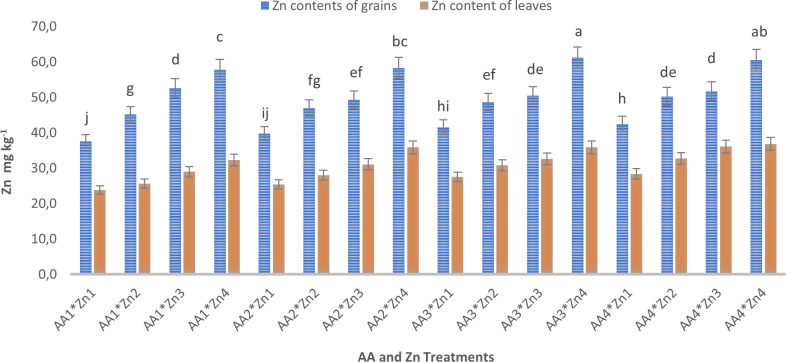
Effect of foliar AA and Zn treatments on Zn contents (mg kg^-1^) of soybeans leaves and grain. The letters on the bars indicate the difference between the mean values.

The effects of foliar AA and Zn treatments on the protein content of soybean grains are presented in **Table 2** and **Figure 5**. This indicated that higher doses of AA resulted in increased grain protein content in soybean grains, a plant known for its high protein content. The highest protein content of grain was observed in A_4_ (44.8%), and a linear relationship was observed between increasing AA doses and grain protein content. The application of Zn, however, did not result in any significant alterations in the protein composition of soybean grains. The effects of foliar AA and Zn treatments on the Zn content of soybean leaves and grains are presented in **Table 2** and **Figure 6**. The findings of this study indicate that foliar treatment with AA and Zn led to a notable increase in the Zn levels of both soybean leaves and grains. These results were found to be statistically significant (p<0.001). The application of AA and Zn increased the zinc (Zn) content in soybean leaves, as well as the average concentration of Zn. The greatest values of Zn concentration 35.2 and 33.6 mg kg^-1^ were notably observed in the maximal dosages of AA_4_ and Zn_4,_ respectively.

The interactions between AA and Zn treatments were found to be statistically significant in grains (p<0.05), and the effective AA*Zn dose was determined from the AA_3_*Zn_4_ treatment (61.2 mg kg^-1^). In terms of the combined effect of AA and Zn treatments, it was determined that both AA and Zn treatments increased the Zn concentration in soybean grains and contributed to Zn biofortification. In terms of the average Zn concentration of grain, it was observed that the maximal dosages (AA_4_ and Zn_4_) provided the highest values (51.3 and 59.5 mg kg^-1^). The changes in Zn concentrations in the leaves and grains of the AA and Zn treatments are presented in **Figure 6**. This study revealed a positive correlation between Zn dose of zinc (Zn) and grain accumulation, indicating a linear increase in grain accumulation with higher dose application of Zn. The application of increasing doses of AA additionally promoted an increase in grain accumulation. A considerable increase in the biofortification effect was observed after the application of maximal dosages of AA and Zn.

## Discussion

The objective of this study was to enhance the nutritional quality of soybean crops using improved fertilization programs, focusing specifically on the foliar application of AA and Zn. The combination of Zn treatment and AA was effective for soybean growth, especially in terms of the number of pods and branches. Correlative results were obtained by Abd El-Aal and Eid (2018), highlighting the favorable impact of foliar amino acid spraying on soybean growth and yield. The results of this study are similarly consistent with the research conducted by Zewail (2014), which indicated that the application of amino acids by foliar approaches enhances the growth attributes of bean plants, including increased plant height and an increased number of branches and leaves per plant. The requirement of amino acid nitrogen is one way to increase the growth and yield of all crops. Nitrogen and/or amino acids are essential components of protein synthesis; they are important due to their widespread use in the biosynthesis of a wide variety of non-protein nitrogenous substances, such as pigments, vitamins, coenzymes, purines, and pyrimidine bases. Several studies have reported that foliar application of amino acids results in increased plant growth, yield, and composition (Kamar and Omar, 1987; El-Shabasi et al., 2005). Zinc is another trace element that is essential to all living organisms. In this study, we identified that zinc application increases the number of branches and pods. The finding was also in partial conformity with the works of Choudhary et al. (2014) and Kanase et al. (2008), who outlined that zinc application increases the number of branches and pods. Similar to our findings, Imsong et al. (2023) outlined that the number of pods was highest at the highest level of zinc and lowest in the control group. Also, the authors noted that elevated zinc fertilization resulted in a more pronounced plant response with enhanced branch numbers. This change in growth parameters may be linked to the involvement of zinc in tryptophan synthesis, nitrogen metabolism, and the production of growth hormones like indole acetic acid (Imsong et al., 2023). The above findings align with the research conducted by Raghuwanshi et al. (2017) and Singh et al. (2017), which stated the enhanced growth characteristics of soybean with the application of zinc.

The foliar application of AA and Zn demonstrated no significant effect on soybean grain yield (**Table 1**). These findings are consistent with those of Enderson et al. (2015), indicating that the foliar applications of B, Cu, Mn, Zn, and their mixture did not result in an increase in soybean grain yield across all 42 sites. Similar findings have been reported in other studies for soybean. For example, Souza et al. (2019) identified no significant effect of Zn and AA foliar fertilization on yield components, and Mallarino et al. (2001) reported that foliar fertilization with various nutrient mixtures, including Zn, resulted in very small and infrequent yield increases. Similar results have also been identified for different crops; Teixeira et al. (2008) found no effect of Zn foliar application on the grain weight of beans grown in soil with a sufficient Zn concentration. Cakmak et al. (2010) reported that foliar Zn treatments did not affect wheat grain yield. These studies support the claim that soybeans are less sensitive to Zn fertilizer than other crops (Sutradhar et al., 2017). The findings contrast with those reported by Vahedi (2011); Tousi et al. (2014), and Teixeira et al. (2018), indicating that foliar application of amino acids improves the yield components of soybean plants.

Foliar AA treatment was found to increase the nitrogen concentration in soybean leaves and grains. Zn treatments were not as effective as increasing the nitrogen concentration in the leaves and grains of soybeans (**Table 2**). Amino acids help increase nitrogen and some nutrients in plants (Liu et al., 2008; Abo Sedera et al., 2010). This might be because amino acids demonstrate chelation properties with nutrients, thereby enhancing the uptake and translocation of these vital elements inside the plant. This phenomenon may be related to the effects on cell membrane permeability, which enhances the efficiency of nutrient absorption (Marschner, 1995). The use of amino acids as nitrogen (N) and carbon (C) sources by plants has been widely documented in studies (Thornton and Robinson, 2005). Furthermore, the application of lithovit at a rate of 500 mg and amino acids at a rate of 4 ml l^-1^ provided the highest values of leaf chemical composition (N, P, K, Ca, Mg %, and Fe ppm) in soybean plants (Abd El-Aal and Eid, 2018). Similarly, the application of amino acids by foliar spraying resulted in an enhancement of nitrogen content in plants compared to that in the control group in another study (Shehata et al., 2011). Several studies have demonstrated the efficiency of amino acid uptake by plants, with the foliar application of amino acids demonstrating promising results (Abdel-Mawgoud et al., 2011; Sadak et al., 2014).

The results of this study indicated that higher doses of AA led to an increase in the protein content of soybean grains. Varying the dosages of Zn, however, did not have a significant impact on the protein content of the soybean grains. The use of amino acids may have favorable effects, which may be attributed to their internal roles as osmoregulatory agents (Treichel, 1975). This is due to their high solubility in water, which leads to an increase in the concentration of osmotic components inside the cells (Abdel-Mawgoud et al., 2011). Suciu et al. (2022) reported that the application of biofertilizers containing amino acids contributed to the protein content of soybeans. Research on the positive effect of biostimulants on the protein content in legume seeds is available in terms of common beans; the treatment of *Fabaceae* plants with biostimulants containing amino acids has resulted in an increase in protein content in the seeds of common beans (Zewail, 2014; Kocira et al., 2017).

Foliar treatments of AA and Zn increased the average Zn concentration of soybean leaves and grains. The most effective dose was obtained from the AA_3_*Zn_4_ treatment in the AA*Zn interaction in grain Zn concentration. Increasing the treatment rates of AA and Zn increased the Zn accumulation in both leaves and grains, and the AA*Zn combination contributed to Zn biofortification, especially in the grains. The application of foliar zinc sulfate was effective in biofortifying winter wheat by increasing the grain Zn from 20 to 30 mg kg^-1^ (Mao et al., 2014). Choudhary et al. (2014) stated that by increasing the Zn doses, the Zn content increased in soybean grain, with the highest Zn content of soybean grain being measured at 57.4 mg kg^-1^. Foliar fertilization with Zn at a ratio of 4 (0, 0.91, 1.82, 2.73, and 6.37 mg kg^-1^) increased plant height, grain mass, and protein content in soybean grains (Oliveira et al., 2018). In addition, Zn is crucial for the function of dehydrogenase, proteinase, RNA enzymes, and chlorophyll synthesis (Hansch and Mendel, 2009). These results can be considered preliminary in establishing the optimal dosage of AA*Zn for future studies aimed at gaining information into these mechanisms.

Foliar application, which contributes to the rapid uptake of nutrients from leaves and minimizes environmental pollution and groundwater contamination (Dehnavard et al., 2017), may be a viable agronomic biofortification practice for zinc at the advanced stages of soybean development. A greenhouse study on soybeans reported that foliar Zn applied at the full-pod stage at low soil Zn concentrations was more effective than other treatments in improving seed Zn concentration (Oliveira et al., 2018). Moreover, considering the low canopy height of soybeans, it allows the use of a ground sprayer (Cuesta et al., 2023).

## Conclusion

In this study, foliar Zn and amino acids, an organic compound and biostimulant, were applied together to soybean to determine plant development and Zn biofortification performance, particularly in the grain. Positive effects of the co-use were observed in some foliar applications. While no effect of foliar treatments was observed on the yield values, the combined treatments demonstrated significant effects on the pod number (AA_2_*Zn_2_) and branch number (AA_4_*Zn_2_). There was a significant combined effect of AA and Zn, where the application of AA_3_*Zn_4_ provided the maximum value for Zn biofortification. In addition to foliar Zn application at later stages of cultivation, the application of materials with organic components (AA, seaweed, etc.) may have increased the availability of Zn by supporting the uptake in leaves and grains.

## Data availability statement

The raw data supporting the conclusions of this article will be made available by the authors, without undue reservation.

## Author contributions

ŞH: Methodology, Writing – original draft. İS: Conceptualization, Formal analysis, Methodology, Writing – original draft. MQ: Writing – review & editing. BG: Methodology, Writing – original draft. SG: Writing – original draft. EY: Formal analysis, Writing – review & editing.

## Supplementary material

Supplementary Figure S1 The mean monthly rainfall, air temperature, and humidity during the growing period.
