# Supplementary figures and images for: The effects of foliar amino acid and Zn applications on agronomic traits and Zn biofortification in soybean (Glycine max L.)
Source: Front Plant Sci. 2024 Apr 15;15:1382397. doi: 10.3389/fpls.2024.1382397 (PMC11056589; doi:10.3389/fpls.2024.1382397)

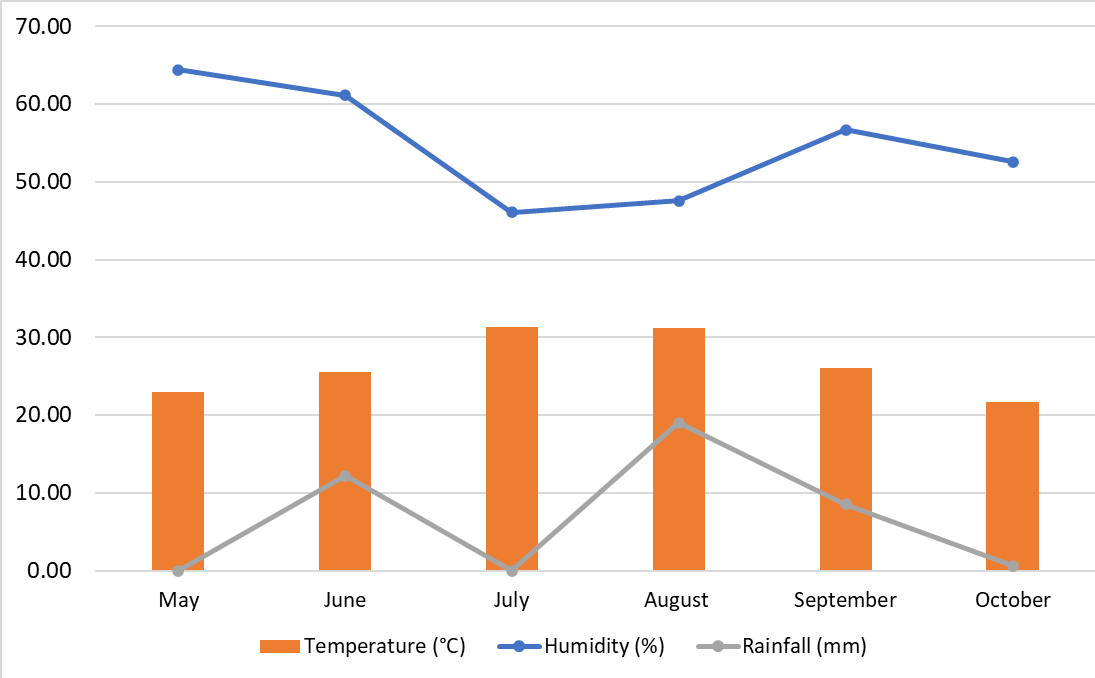

Supplement: Supplementary Figure S1 — The mean monthly rainfall, air temperature, and humidity during the growing period. [file Image_1.tif]
