# Supplementary material for: The effects of foliar amino acid and Zn applications on agronomic traits and Zn biofortification in soybean (Glycine max L.)
Source: Front Plant Sci. 2024 Apr 15;15:1382397. doi: 10.3389/fpls.2024.1382397 (PMC11056589; doi:10.3389/fpls.2024.1382397)
Supplement: Supplementary file 2 [file Table_1.docx]

**Table S1**. The experimental soil analysis results

| **Parameters** | **Units** | **Value** |
| --- | --- | --- |
| Total CaCO_3_ | % | 20.9 |
| Organic matter | % | 1.23 |
| pH |  | 7.7 |
| EC | % | 0.045 |
| Texture |  | Loamy |
| Total Nitrogen (N) | % | 0.089 |
| Available Phosphorus (P) | mg kg^-1^ | 7.04 |
| Extractable Potassium (K) | mg kg^-1^ | 321.1 |
| Extractable Calcium (Ca) | mg kg^-1^ | 6950,8 |
| Extractable Magnessium (Mg) | mg kg^-1^ | 352,2 |
| Available Iron (Fe) | mg kg^-1^ | 2.58 |
| Available Zinc (Zn) | mg kg^-1^ | 0.82 |
| Available Manganese (Mn) | mg kg^-1^ | 5.51 |
| Available Copper (Cu) | mg kg^-1^ | 0.88 |
